# Gastric undifferentiated carcinoma, INI1-negative

**DOI:** 10.4322/acr.2021.408

**Published:** 2022-11-03

**Authors:** Gabriele Gaggero, Luca Carlin, Margherita Concardi, Marta Ingaliso, Davide Taietti

**Affiliations:** 1 IRCCS Ospedale Policlinico San Martino, U.O. Anatomia Patologica Ospedaliera, Genova, Italy; 2 Università di Genova, Scuola di Scienze Mediche e Farmaceutiche, Dipartimento di Scienze Chirurgiche e Diagnostiche integrate (DISC), Divisione di Anatomia Patologica, Genova, Italy; 3 Azienda Socio Sanitaria Territoriale (ASST) Ospedale Maggiore, Unità di Anatomia Patologica, Crema, Italy

**Keywords:** undifferentiated carcinoma, gastric, stomach, INI1

Among undifferentiated carcinomas of the digestive system (DS-UC), undifferentiated gastric carcinoma (GUC) is one of the rarest epithelial malignancies of the stomach, accounting for 0.1-0.3% of all gastric carcinomas.^1^

The limited data on DS-UC/GUC describe them as aggressive, usually at an advanced stage and with a marked tendency to lymph node metastasis, even when still intramucosal.^1^^,^^2^ While for other gastric neoplasms (both epithelial and lymphoid), there are definite etiological correlations, i.e., with infectious agents (*Helicobacter pylori* or EBV), both the etiology and the molecular pathways of GUC remain largely unknown: the few data available suggest dedifferentiation from a glandular neoplasm (indeed, cases are described in which areas of well-differentiated gastric adenocarcinoma coexist with GUC); however, this assumption is not always proved, neither from a morphological nor a molecular point of view. Other data indicate a correlation between the alteration of molecular pathways linked to the SWI/SNF chromatin-remodeling complex, with loss mainly of SMARCB1 (INI1) and some histological forms of GUC, in particular the rhabdoid histotype.^3^^-^6 Several variants of DS-UC/GUC are described: anaplastic with marked cellular pleomorphism, sarcomatoid, with osteoclast-like giant cells and lymphoepithelioma-like;^1^ however, the aforementioned INI1-negative rhabdoid is the most commonly reported histo-molecular variant, both in the stomach and in the whole digestive system.^3^^-^6 It is worth mentioning that the most frequent location of INI1-negative tumors is in the head and neck region, while gastroenteric ones are extremely rare.^7^

The photo above refers to an 84-year-old woman found unconscious at home due to an unknown cause. On admission to the hospital, jaundice and acute liver failure were observed together with the presence of a mesogastric mass strongly suspected on radiology to be neoplastic. Before any further diagnostic/therapeutic steps could be taken, death occurred.

The autopsy reveals the presence of a large antral neoplasm with a maximum diameter of 5.5 cm (Figure 1A), with numerous perigastric and peripancreatic enlarged lymph nodes of metastatic significance. The voluminous neoplasia causes ab-extrinsic compression of the extrahepatic biliary tract, macroscopically justifying jaundice found at admission. The liver shows multiple intraparenchymal whitish dots, while the other organs examined, both abdominal (intra- and extraperitoneal) and thoracic and intracranial, show no significant macroscopic lesions.

**Figure 1:**
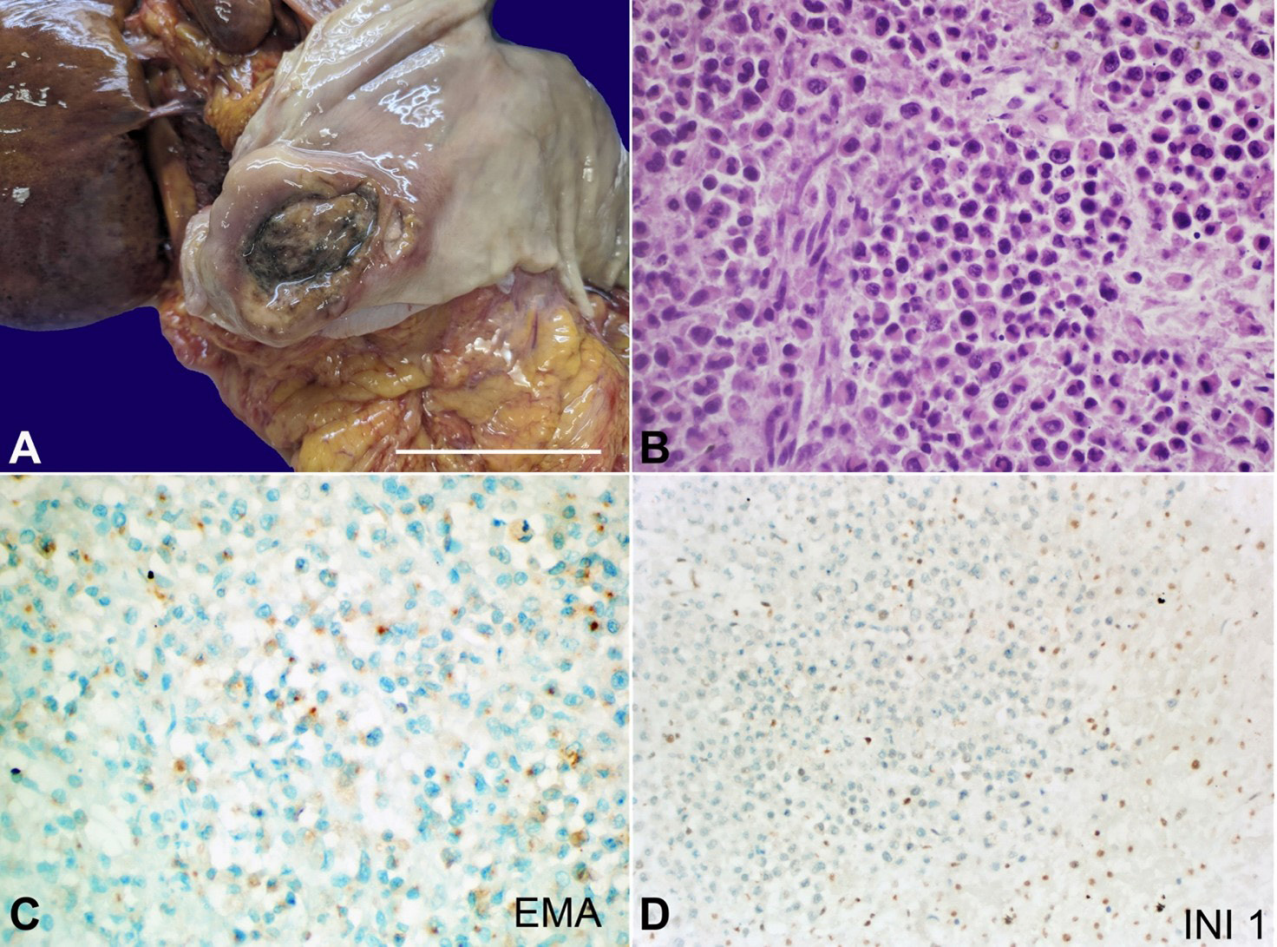
**A** - macroscopic view of the gastric mucosa with a large centrally depressed and ulcerated antral neoplasm (scale bar = 5 cm); **B** - photomicrograph of the gastric lesion, showing the presence of a neoplasm with atypical non-cohesive, round/rhabdoid cells, with scant cytoplasm and frequent mitotic figures (H&E, 60x); **C** - immunohistochemistry, showing focal positivity ‘dot-like’ for epithelial membrane antigen (EMA); **D** - immunohistochemistry of liver metastases, indicating loss of expression for INI1 in the neoplastic cells (on the left), whereas INI1 expression is retained in the nuclei of hepatocytes (on the right).

Microscopically, the neoplasm is composed of medium-sized undifferentiated/rhabdoid cells with numerous mitoses, intratumoral necrosis, and intermingled lymphocytes (Figure 1B). The neoplasm infiltrates the gastric wall also with serosa involvement and lymphatic and hematic embolization, which corresponds histologically to widespread lymph node and hepatic metastatic localization (the latter also explains the liver failure clinically observed).

Immunohistochemistry shows a focal positivity for MNF116 broad-spectrum cytokeratins and a dot-like expression of EMA (Figure 1C) and Vimentin. At the same time, it is negative for muscle markers (Actin, Desmin, Myogenin, Calponin, and Caldesmon), neuroendocrine markers (Chromogranin, Synaptophysin, NSE, and CD56), hematolymphoid lineage markers (CD45, CD3, CD20, CD79alpha, CD68, CD138, MPO) and melanocytic markers (S100, Melan-A, HMB-45). EBV, CD99, CD117, SALL4, and Glypican3 were also negative. Finally, the absence of expression of INI1 (Figure 1D) is noteworthy.

From a strictly microscopic point of view, the features of the neoplastic cells suggest the differential diagnosis between a GUC, an EBV-associated carcinoma with lymphoid stroma, an aggressive lymphoma, a metastatic melanoma, a germ cell neoplasm, a PEComa, and a rhabdomyosarcoma (or other types of sarcomas with epithelioid/rhabdoid pattern).^1^ Based on the overall immunomorphological data, which allows us to exclude the other aforementioned hypotheses, we make the final diagnosis of GUC with rhabdoid morphological aspects and, according to the literature, lack of immunophenotypic expression for INI1.

## References

[1] Agaimy A, Lokuhetty D, White V, Watanabe R, Cree I (2019). WHO classification of tumours of the digestive system.

[2] Nakamura R, Omori T, Mayanagi S (2019). Risk of lymph node metastasis in undifferentiated-type mucosal gastric carcinoma. World J Surg Oncol.

[3] Tessier-Cloutier B, Schaeffer DF, Bacani J (2020). Loss of switch/sucrose non-fermenting complex protein expression in undifferentiated gastrointestinal and pancreatic carcinomas. Histopathology.

[4] Agaimy A, Daum O, Märkl B, Lichtmannegger I, Michal M, Hartmann A (2016). SWI/SNF complex-deficient undifferentiated/rhabdoid carcinomas of the gastrointestinal tract: a series of 13 cases highlighting mutually exclusive loss of SMARCA4 and SMARCA2 and frequent co-inactivation of SMARCB1 and SMARCA2. Am J Surg Pathol.

[5] Horton RK, Ahadi M, Gill AJ (2021). SMARCA4/SMARCA2-deficient carcinoma of the esophagus and gastroesophageal junction. Am J Surg Pathol.

[6] Agaimy A, Rau TT, Hartmann A, Stoehr R (2014). SMARCB1 (INI1)-negative rhabdoid carcinomas of the gastrointestinal tract: clinicopathologic and molecular study of a highly aggressive variant with literature review. Am J Surg Pathol.

[7] Agaimy A, Bishop JA (2021). SWI/SNF-deficient head and neck neoplasms: an overview. Semin Diagn Pathol.

